#  Antimicrobial peptide production with *Corynebacterium glutamicum* on lignocellulosic side streams

**DOI:** 10.1186/s13068-024-02587-1

**Published:** 2024-12-18

**Authors:** Daniel Waldschitz, Mark-Richard Neudert, Jakob Kitzmüller, Jae Hwi Bong, Yannick Bus, Eva Maria Karner, Peter Sinner, Oliver Spadiut

**Affiliations:** Research Group Bioprocess Technology, Institute of Chemical, Environmental and Bioscience Engineering, TU Wien, Gumpendorferstraße 1A, Vienna, A-1060 Austria

**Keywords:** Lignocellulosic feedstock, Spent sulfite liquor, Antimicrobial peptide, Recombinant production, Pediocin PA-1, *Corynebacterium glutamicum*

## Abstract

****Background**:**

Biorefineries usually focus on the production of low-value commodities, such as bioethanol, platform chemicals or single cell protein. Shifting production to bioactive compounds, such as antimicrobial peptides, could provide an opportunity to increase the economic viability of biorefineries.

****Results**:**

Recombinant production of the antimicrobial peptide pediocin PA-1 in *Corynebacterium glutamicum* was transferred from yeast extract-based media to minimal media based on lignocellulosic spent sulfite liquor. Induced batch, fed batch, and extended batch process modes were compared for highest pediocin PA-1 production.

****Conclusion**:**

For pediocin PA-1 production on lignocellulosic residues, extended batch cultivation was identified as the optimal process mode, producing up to $\simeq$ 104 mg/L active pediocin PA-1. Moreover, the production of pediocin PA-1 on this sustainable second generation resource exceeded its state-of-the-art production on yeast extract-based media $\simeq$ 1.5-fold.

**Supplementary Information:**

The online version contains supplementary material available at 10.1186/s13068-024-02587-1.

## Background

Biorefineries have become a poster-child example for reaching the sustainable development goals outlined by regulatory authorities [1]. Still, the development of new lignocellulosic biorefinery projects remains below expectations and many of the existing biorefineries still struggle with economic viability. The valorization of lignocellulosic biomass (LBM) presents a number of challenges. For agricultural residues, seasonal fluctuations in the availability impact commercialization, whereas in common side streams from the forestry industry, high concentrations of inhibitory compounds, such as furfural and 5-hydroxymethylfurfural (HMF), are present [2]. The lion-share of biorefinery production is bioethanol, which has a high annual demand, but is a low-priced commodity [3]. Extensive funding is being provided for the production of alternative solvents, often grouped under term “acetone, butanol, ethanol (ABE) fermentation”. However, bioethanol is typically produced under non-sterile conditions at acidic pH, with native yeasts, such as *Saccharomyces cerevisiae* [4]. Instead, ABE production requires genetically modified organisms (GMO), sterile conditions, and the use of selection markers to ensure genetic stability. Furthermore, complex and expensive media components, such as yeast extract, are added to LBM hydrolyzes to boost productivity [5]. Thus, manufacturing costs are high and ABE production in biorefineries is often outmatched by fossil fuel based production [6].

An alternative to ABE production is the production of peptides and proteins. Among the first industrialized bioprocesses was the production of single cell protein (SCP) using organisms such as *Paecilomyces variotii* [7], *Candida utilis* [8] as well as mixed cultures [9] from spent sulfite liquor (SSL), a commercial side stream from the pulp and paper mills. However, since SCP is a low-cost product, the production of more valuable bioactive compounds for feed, such as antimicrobial peptides (AMPs), is a highly interesting alternative. The market for such feed and food preservatives exceeded USD 1.8 billion and was projected to grow between 4.3% and 23.3% per annum for the next decade [10]. AMPs are commonly commercially available as freeze-dried preparations and ferments for feed and food applications, with only the AMP nisin, registered in the EU as E234, being sold as pure isolate as a food additive [10]. Further, the lignosulfonates remaining from SSL after removal of the sugars by fermentation are considered as safe animal feed by regulatory authorities, such as the European Food Safety Authority (EFSA) [11]. Thus, the switch to SSL-based production does not strictly entail downstream development, while higher retail prices could cover the increased production costs (e.g., GMO requirements). One organism commonly used for the production of functional feed additives is *Corynebacterium glutamicum* [12] with proven capabilities of recombinant production of AMPs, such as nisin [13], garvicin Q [14] as well as pediocin PA1 [15]. In addition to being the industrial workhorse for amino acid production, it has been extensively genetically modified for the uptake of multiple carbon sources [16]. Furthermore, *C. glutamicum* possesses specialized genes for natural resistance to most inhibitors present in LBM hydrolysates and the ability to grow on inexpensive, ultra-filtered spent sulfite liquor (UF-SSL) based minimal media [17]. Therefore, it is a highly promising host organism for the production of AMP on LBM-based media.

## Results and discussion

### Specific challenges in AMP production

The state-of-the-art product quantification method for AMP, such as pediocin PA-1, is by measuring growth inhibition (antimicrobial activity) against the target organism [18], rather than product concentration. This method is preferred due to its high selectivity and sensitivity and results are usually expressed in biological activity per mL sample (BU/mL) instead of mg/L. However, pediocin PA-1 is sensitive to oxidation, resulting in a loss of activity upon oxidation [15]. The production of active product is desired, but aerobic conditions are required for production. Thus, the antimicrobial activity (A) does not represent the product concentration (P) $A~\ne ~P$, and the volumetric (r) and specific (q) rates depend on both production and oxidation $r_A/q_A~=~r_P/q_P~-~r_{OX}$ where only A is quantified. For comparison to product concentration, as a common performance indicator for other products, a factor of $\simeq$ 2,050 BU/ml per mg/L purified active pediocin PA-1 was established in literature [15, 19] and applied in the present study (verified with a standard of known concentration). However, antimicrobial activity, not product concentration, was quantified for all samples, with concentration equivalents provided only for better comparability with other products. Data regarding the verification that the antimicrobial assay is not impeded by a changing sample matrix environment between media are provided in Additional file 1 for interested readers. Moreover, all matrix-based influences diminish with increased sample activity, as matrix-based effects decrease faster with dilution than the highly specific attack of the pediocin PA-1 on the indicator organism.

**Fig. 1:**
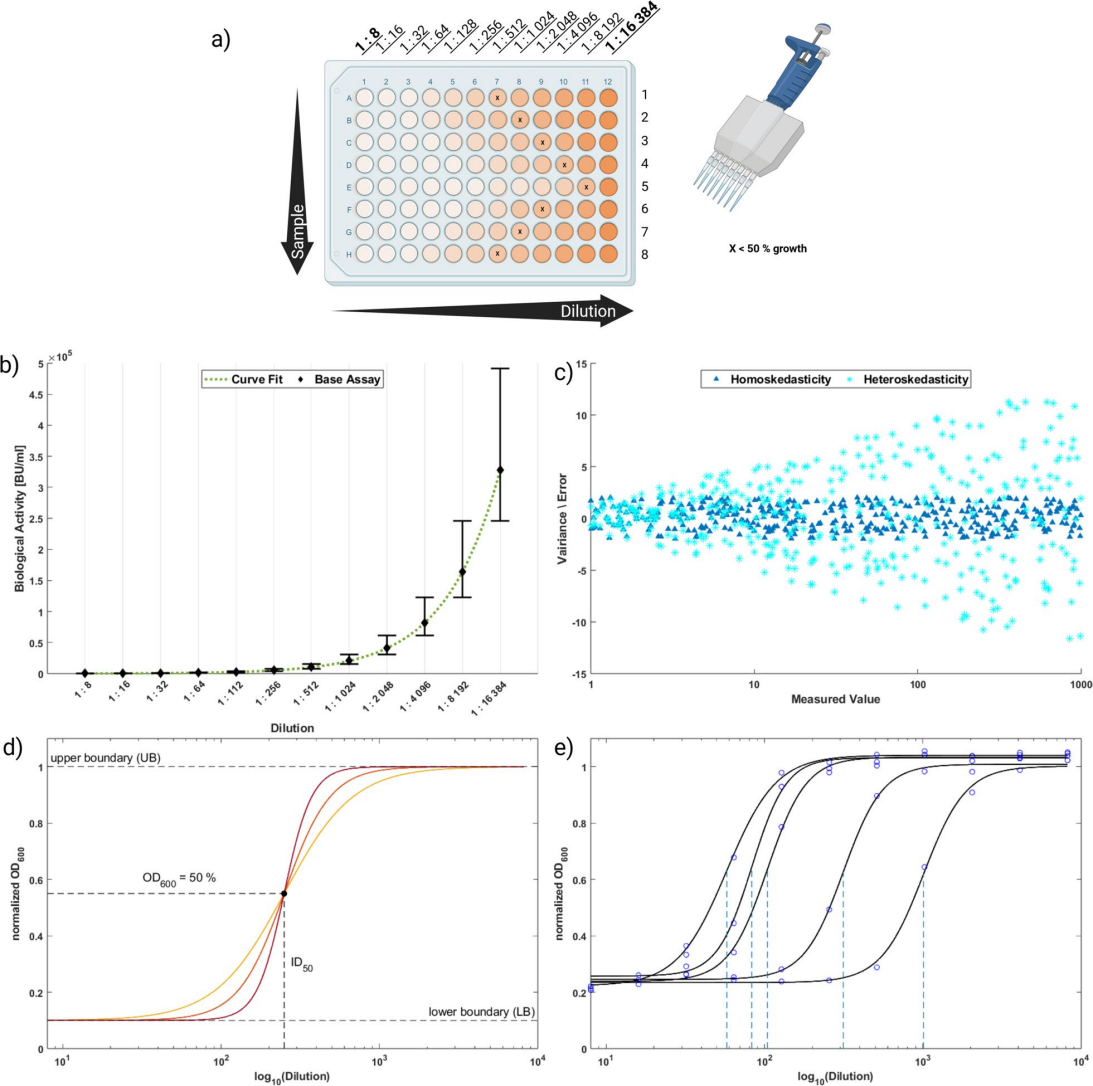
Illustration of the growth inhibition assay (**a**). Calculated antimicrobial activity (**b**) directly from the assay including error bars (black diamonds) and curve fit with unknown error distribution (dotted line). Example of homoscedastic (blue triangles) and heteroscedastic (cyan asterisk) data (**c**). The dose–response curve and the corresponding parameters according to equation 1 for different curvatures are shown in color (**d**), with the relationship between the half growth of the indicator assay and the inflection point shown. Example of samples with increasing activity (**e**) and the resulting calculated dilution at half growth is shown (dashed line)

The primary goal of this study was to gain process knowledge of AMP production on lignocellulosic residues in order to increase the antimicrobial activity obtained. For such purposes, design of experiment (DoE) approaches evaluated by linear regression are commonly used in biotechnology [20]. However, the used state-of-the-art growth inhibition assay [18] presents two challenges. The assay is based on a serial twofold dilution of AMP against a constant target organism concentration on 96-well plates (from 1:8 to 1:16,384) (for illustration see Fig. 1). The well with the lowest dilution, where less than half of the growth of the indicator organism occurs compared to a blank, is used to determine the antimicrobial activity of the sample. This methodology causes two drawbacks. First, the difference in activity between two successive dilutions doubles with each dilution step (resolution halves), and thus providing less accurate results at higher activities. As a countermeasure, a dose–response curve fit [21] was used to calculate more accurate results (improved resolution) as suggested for AMP [19]. Thereby, the optical density of the target organism in each well was used to calculate the inflection point where $50\%$ growth occurs instead of using just the well with $<50\%$ growth. Second, the measurement error per well increases exponentially (low precision), leading to heteroscedasticity (unequal variance over the analyzed measurement range). However, homoscedasticity, which describes a constant variance (measurement error) over the analyzed measurement range, is an assumption of ordinary linear regression [22]. While more advanced options (weighting, transformation [22]) and methods (maximum likelihood [23], Bayesian estimation [24]) exist to deal with non-homoscedastic data, the curve fit simultaneously limits the heteroscedasticity of the assay. Thus, advanced alternatives are only necessary when the desired goals of gaining process understanding and increasing maximum activity cannot be achieved otherwise. Hence, a combination of process optimization using a DoE linear regression approach based on activity data from a dose–response curve fit was chosen as strategy for this study.

### Transferability between media

In recent years, the production of pediocin PA-1 by *Corynebacterium glutamicum* was established by [15] and improved by [19] on complex yeast extract-based medium. Here a brief summary of highlights from their studies is provided and the transferability to lignocellulosic minimal medium-based production is discussed. [15] first established pediocin PA-1 production using *Corynebacterium glutamicum* CR099 on a yeast extract medium (2TY complex medium + salts and vitamins from CGXII defined media) and achieved a maximum of 20,480 BU/ml $\simeq$ 10 mg/L active pediocin PA-1 using a fed batch process. Production improvements in bioprocess development were discussed, such as increasing the biomass concentration and adjusting the oxygen supply during production to prevent product oxidation. [19] further analyzed the optimal $dO_2$ levels ($dO_2$ of 30/10/2.5 %) for pediocin PA-1 production using a complex yeast extract-based medium and found a $dO_2$ of 2.5 % to be optimal during production. In addition, pediocin PA-1 production was enhanced at acidic pH values (best pH 5.9$-$6.0) and at increased concentrations of bivalent ions such as $\hbox {Ca}^{2+}$ (best 2 g/L $\hbox {Ca}^{2+}$). As hypothesis, the interaction of the cationic peptide with the negatively charged residues of the cell wall of *Corynebacterium glutamicum* has been proposed. Thus, both the presence of $\hbox {H}^+$ and bivalent $\hbox {Ca}^{2+}$ ions were hypothesized to reduce the absorption of pediocin PA-1 onto the cell surface. A maximum of 135,700 BU/mL $\simeq$ 66 mg/L active pediocin PA-1 was obtained using a delayed induced batch (shift from growth to production conditions after 4 h) process mode [19]. In summary, biomass concentration, $dO_2$ levels, pH and bivalent ion concentration were highlighted for their impact on production utilizing complex yeast extract-based media.

In this study, in contrast to prior studies, no yeast extract was used for bioreactor production and UF-SSL was the only complex component used in the minimal medium. UF-SSL contains significant amounts of lignosulfonates, which are water-soluble anionic polymers resulting from the sulfite pulping process [25]. Among other applications, lignosulfonates are used industrially for protein/peptide purification by precipitation, with patents filed for both enzyme [26] and AMP [27] recovery. A reversible complex formation between the lignosulfonates and the protein/peptide surfaces (complex formed below protein pI and dissolved above protein pI) was proposed as the cause of the precipitation and employed for protein recovery. The reversible complex formation was reproduced using UF-SSL and pediocin PA-1 standard (see Additional file 1), with centrifugation conditions adjusted for all experiments to ensure minimal product loss due to centrifugation for cell removal. The beneficial effects of increased biomass concentration and lower pH on pediocin PA-1 were considered to be transferable from yeast extract to UF-SSL-based medium. However, the UF-SSL used in this study contained 14 g/L $\hbox {Ca}^{2+}$, exceeding the recommended optimal $\hbox {Ca}^{2+}$ concentrations by a factor of 2 (in the 25 % dilution used as part of the minimal medium). Therefore, the adjustment of the bivalent ion concentration in the medium is not one-to-one transferable between media. The concentration of soluble bivalent ions in the UF-SSL minimal medium is influenced by two factors. First, the addition of phosphorus to UF-SSL-based minimal medium results in precipitation of calcium as well as other cations present in UF-SSL [17]. Second, the amount of precipitation is affected by pH in addition to phosphate concentration. Therefore, pH and the amount of $\hbox {PO}_4$ added were used instead of the calcium concentration in the medium to evaluate the effect of bivalent ion concentration on pediocin PA-1 production. Similarly, the transferability of adjusted $dO_2$ levels on yeast extract-based medium was questioned, with initial screening experiments showing no significant impact of the $dO_2$ level applied on the maximum antimicrobial activity. Initially, the described attachment of lignosulfonates to protein/peptide surfaces was hypothesized to reduce the accessibility of the oxidation site (L-methionine residue at position 31 [19]) to dissolved oxygen (similar to the reduced antimicrobial activity in the presence of lignosulfonates at low dilutions). In contrast, pediocin PA-1 oxidation was found to be higher in UF-SSL-based minimal medium than yeast extract-based medium (see Additional file 1), indicating that factors other than oxidation may be more prevalent. However, further research into the exact causes of this discrepancy would be necessary with investigations into the exact metabolic impact in both media but was beyond the scope of this study. Therefore, the $dO_2$ levels were set to 15% during dedicated production phases to limit oxidation potential while imposing less respiratory constraints (2.5% reported as optimal on yeast extract [19]) on the more challenging substrate.

### Induced batch

**Fig. 2:**
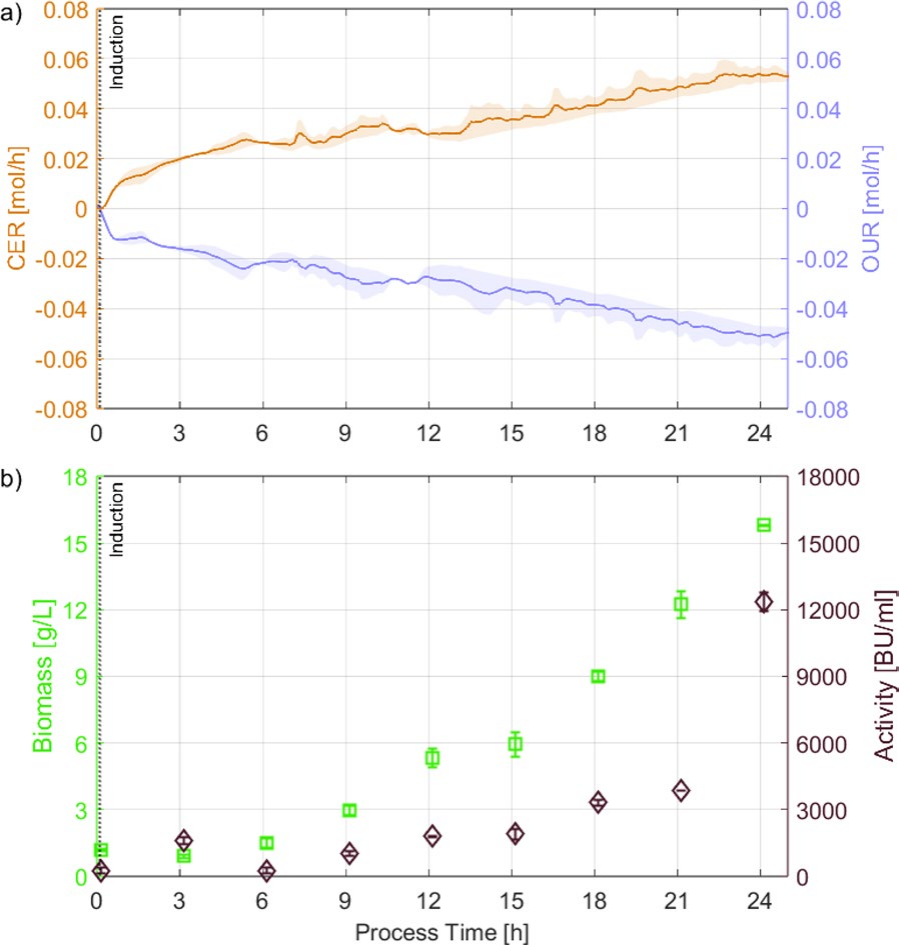
**a** Mean and standard deviation of CER (orange line), OUR (blue line);** b** biomass (green squares) and product activity (violet diamonds) of induced batch cultures of *C. glutamicum* on UF-SSL minimal medium. Minimal medium and growth conditions (except induction) were taken from [17], for more details regarding the substrate utilization readers are refereed there. Error bars and shading represent biological variation between 2 replicates, not technical replicates of measurements

An induced batch is the simplest process mode for recombinant production. However, optimal conditions for growth and recombinant production might not overlap. Contrary to optimal production reported on complex medium (pH 5.9$-$6.0, $dO_2$ 2.5% [19]), optimal batch growth of uninduced *C. glutamicum* on UF-SSL was reported at pH a of 7.0 compared and a $dO_2$ of 30% [17]. Moreover, the shortest doubling times for *C. glutamicum* were reported to be between pH 6.5 and 8.0 with growth limits at pH 5.5 and 9.5 [28], placing the reported pH optima for production close to the physiological capabilities of the organism. Since low $dO_2$ levels impose further respiratory constrains on the cells, process conditions optimized for growth [pH 7.0, $dO_2$ 30 %, C/P ratio 1:30] were chosen to establish the baseline production of pediocin PA-1 in an induced batch (see Fig. 2). Since the cells detoxify high concentrations of inhibitory compounds, such as furfural and HMF before proliferation [17], no biomass growth was observed in the first sample after inoculation/induction (3 h). In contrast, the antimicrobial activity obtained at 3 h decreased thereafter and was only reached at 12 h with $\simeq$ 6-fold higher biomass concentrations. Towards the end of the process, between samples at 21 h and 24 h, the carbon dioxide evolution rate (CER) and the oxygen uptake rate (OUR) stagnated, indicating that growth rapidly decreased before the final sample. Similar to the low growth coinciding with an uptick in antimicrobial activity between 0–3 h, antimicrobial activity showed the greatest uptick between 21–24 h. Therefore, high biomass growth rates were hypothesized to suppress pediocin PA-1 production. Under these conditions, a maximum antimicrobial activity of 12,357 ± 1,119 BU/mL was achieved after 24 h. While it was promising that pediocin PA-1 could be recombinantly produced on UF-SSL, it was $\simeq$ tenfold lower than values reported on a yeast extract-based medium in fed batch, highlighting the need for separate growth and production phases to achieve higher production [15].

### Fed batch

In order to screen for improved production conditions, a fed batch process consisting of an uninduced batch phase and an induced feeding phase using a design of experiment approach was chosen. The design space (DS) for pH was set based on the reported pH for growth on UF-SSL minimal medium (pH 7.0 [17]) and the optimal production on complex medium (pH 6.0 [19]). Varying $\hbox {PO}_4$ availability in the feeding phase was done by adjusting the carbon to phosphate (C/P) ratio between the UF-SSL feed and the separate NP feed. $dO_2$ was decreased from 30 % to 15 % after induction to reduce potential for pediocin PA-1 oxidation, while not imposing respiratory constraints on the cells.

**Fig. 3:**
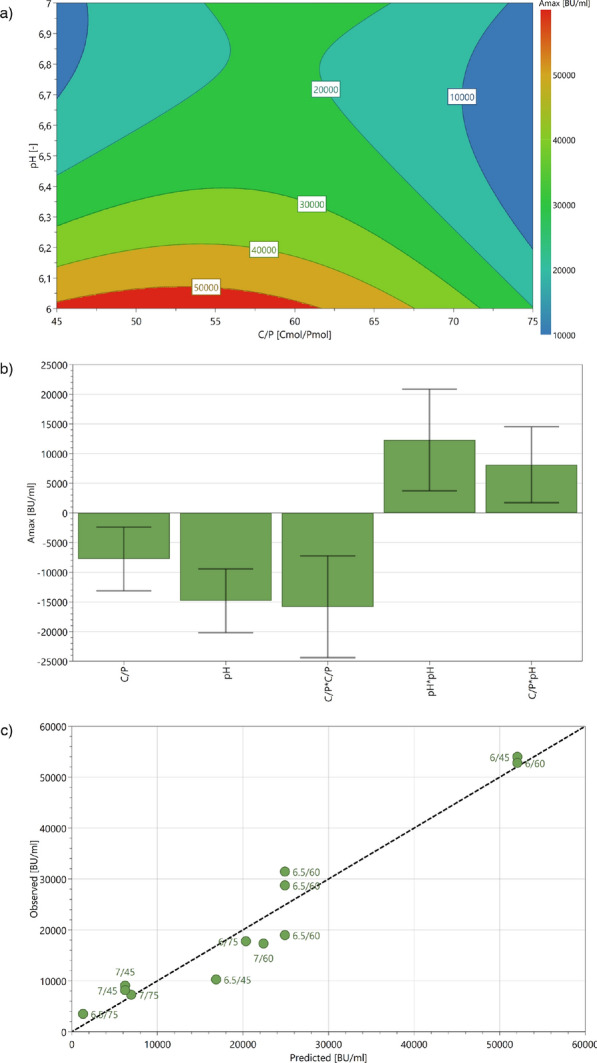
The DS of the DoE was analyzed using an MLR model, with the surface plot (**a**), response terms (**b**) and observed versus predicted values (**c**). Both quadratic response terms [pH*pH and C/P*C/P] as well as the interaction term [pH*C/P] were shown to have a significant influence on the maximum pediocin PA-1 activity

To generate a multiple linear regression (MLR) model, the maximum pediocin PA-1 antimicrobial activity obtained was selected as the response. Significant quadratic effects for pH and C/P were detected. Furthermore, the impact of the interaction between pH and C/P on both precipitation and production was ascertained. However, some terms were just above the threshold of significance and the variability of all terms was high. Two potential causes for the high variability of the results were hypothesized. First, while the curve fit used to calculate more accurate results changes the exponential heteroscedasticity stemming from the assay, it may not generate a fully homoscedastic distribution. Thus, the MLR may suffer from unequal error distribution of the single repetition experiments at the edge of the DS ($\simeq$ 1:2,048 dilution in the assay results in $\ge$ 50% growth reduction of the *Listeria spp.* for the highest samples). Second, similar to the CER and OUR profiles of the induced batch cultures, a stagnation was observed before 24 h (start of feeding). This stall in exponential growth could indicate a decrease in cell viability leading to higher variability between replicates. In summary, despite the high variability (see Figure 3), a statistically significant model was generated (coefficient of determination $R^2$ = 0.94; goodness of fit $Q^2$ = 0.78; detailed statistical data can be found in Additional file 1). Maximum antimicrobial activity was observed at the edge of the DS pH 6.0 and C/P 1:45, representing a $\simeq$ fourfold increase over the induced batch. Thereby, both primary goals of advancing process understanding and increasing activity using lignocellulosic feedstocks were achieved.

**Fig. 4:**
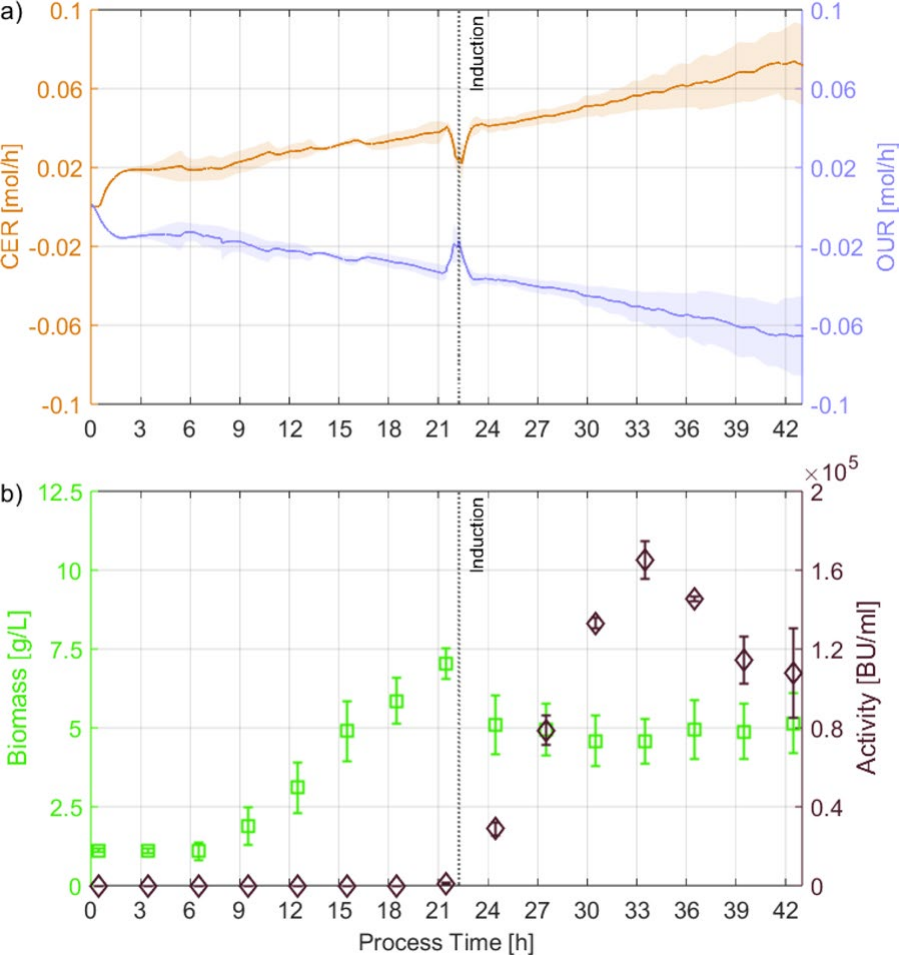
**a** Mean and standard deviation of CER (orange line), OUR (blue line),** b** biomass (green squares) and product activity (violet diamonds) of *C. glutamicum* on UF-SSL minimal medium from DoE centerpoint cultures using an induced fed batch process started after 21 h of uninduced batch. Error bars and shading represent biological variation between 3 replicates, not technical replicates of measurements

To continue, further DoEs were performed to compensate for the hypothesized decrease in viability and to ensure a starting point with a reduced spread for the induced feeding phase. Therefore, induction was performed within the exponential growth phase of the culture [21 h], where no stagnation of the CER and OUR was observed in the previous experiments. Since the observed maximum was found at the edge of the DS, two additional DoEs were performed with the earlier induction. One with exactly the same DS as the 24 h DoE, in order to have a one-to-one comparison, and one where the DS was adjusted based on the results of the 24 h DoE to check whether the optimum was within the DS of the 24 h DoE. Regarding the factor pH, the highest antimicrobial activity was observed around pH 6.0, similar to results reported in literature [19]. Hence, lower pH values were included in the DS of the second DoE [pH 6.5/6.0/5.5]. Since no growth of *C. glutamicum* was reported below pH 5.5 [28], no further reduction below of pH 5.5 was considered. Regarding the factor C/P, it was observed that the most favorable C/P ratio for production shifted to higher $\hbox {PO}_4$ concentrations at lower pH values in the 24 h DoE. Consequently, the DS for C/P was adjusted by analyzing higher $\hbox {PO}_4$ to match the lower pH values used for the updated DS [C/P 1:30/45/60]. Using the earlier induction and feeding start, maximum activities were increased for all experiments compared to the 24 h DoE. The highest maximum activity was measured with 178,949 ± 16,613 BU/mL at pH 6.0 and C/P 1:45 (see Fig. 4). This amounts to a 3.4-fold increase in maximum antimicrobial activity achieved only by shifting the induction time by 3 h. All experiments showed a decrease in activity after the maximum was reached, indicating that product oxidation started to exceed product formation ($r_P/q_P~<~r_{OX}~\Rightarrow ~-r_A/q_A$) at later process times. However, no MLR model with significant terms could be generated for both 21 h DoEs. The gradual change in behavior from $r_P/q_P~>~r_{OX}$ (21–33 h) to $r_P/q_P~<~r_{OX}$ (33–42 h) may have resulted in the peak antimicrobial activity not coinciding with a sample of the fixed sampling interval. Therefore, the MLR was subjected to a higher degree of uncertainty while employing the maximum antimicrobial activity as a response. Higher activities thereby decreased both the accuracy and precision of the assay ($\simeq$ 1:8,192 dilution in the assay results in $\ge$ 50% growth reduction of the *Listeria spp.* for the highest samples). For example, the standard deviation of the biological triplicates of the 21 h DoE center point (16,613 BU/mL) was greater than the maximum activity of the induced batch (12,357 BU/mL). While a statistical evaluation of the DS was hindered by the analytical variability of the growth inhibition assay, the results obtained exceeded the production on yeast extract-based media $\simeq$ 1.3-fold. This was achieved using higher $dO_2$ levels (15% $dO_2$ compared to 2.5%) which impose less respiratory constraints on the cells. Hence, process understanding was demonstrated, as the obtained maximum match between the 24 h DoE and both 21 h DoEs (pH 6.0; C/P 1:45) and maximum antimicrobial activity was pushed past the literature reference on yeast extract-based media, utilizing cheap lignocellulosic feedstocks instead. Therefore, employing more advanced statistical methods than ordinary linear regression was not deemed necessary and the focus was set on improving the understanding of the effect of the shift to earlier induction.

In single-substrate cultivations, feeding is typically started upon depletion of the single substrate in the medium. For multi-substrate systems, such as UF-SSL, certain primary substrates, especially glucose, are depleted before other secondary substrates (e.g., mannose) are metabolized, and the substrate with the lowest affinity (e.g., xylose) might take exceedingly long until depletion. In this case, growth stagnation or even cell starvation and partial lysis can occur before depletion of all substrates. Feeding after a pre-determined batch duration will result in an excess of secondary substrate during the early feeding phase until an equilibrium is reached. The more excess secondary substrates are available at the start of feeding, the more similar the conditions are to a batch extension. Since an increase in product activity was observed for more secondary substrate overhang, an extended batch approach was compared to the fed batch approach.

### Extended batch

Extended batch processes are preferable for industrial settings due to their reduced complexity and ease of operation. Hence, the hypothesized benefit of substrate unlimited conditions was tested while applying the process understanding gained from the DoE approach. Cultivation conditions of the fed batch with the highest maximum product activity [uninduced: pH 7.0, $dO_2$ 30%, 21 h; induced: pH 6.0, $dO_2$ 15%] were used, but instead of feeding 1 L UF-SSL minimal medium, using an exponential function, the feed was added completely after 21 h within $\le$ 90 s. In addition, the NP feed was simplified [C/P 1:45, 200 mL, constant 6 mL/h] to further reduce the complexity of the process and thus promote industrial implementability. Furthermore, for lowering the pH from 7.0 to 6.0 upon induction, the UF-SSL-based feed with pH 4.3$-$4.5 was used to reduce acid consumption. This reduced the potential commercial cost of production both in terms of lower capital costs, due to reduced process complexity, and lower operating costs, due to reduced resource use, beyond what is already contributed by the switch from yeast extract to lignocellulosic production.

**Fig. 5:**
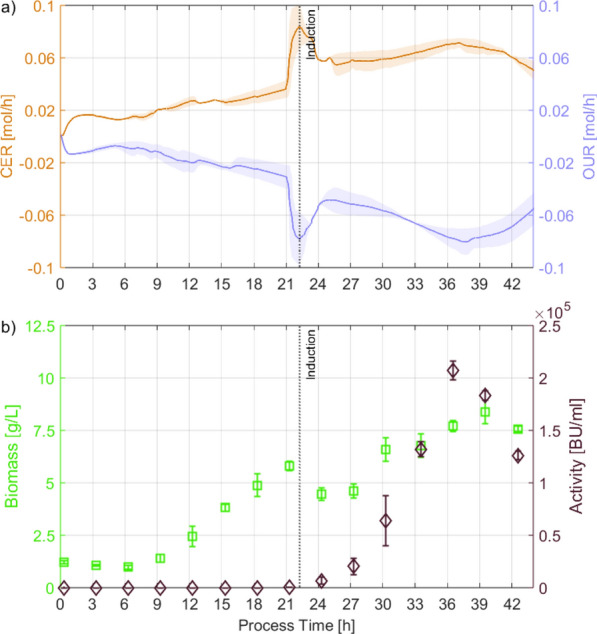
**a** Mean and standard deviation of CER (orange line), OUR (blue line);** b** biomass (green squares) and product activity (violet diamonds) of *C. glutamicum* on UF-SSL minimal medium from uninduced batch cultures extended and induced after 21 h. Error bars and shading represent biological variation between 2 replicates, not technical replicates of measurements

Maximum antimicrobial activity of 206,978 ± 24,717 BU/mL was achieved for the extended batch process mode (see Fig. 5). In contrast to the induced batch, the induction was performed at high cell densities upon batch extension, resulting in an $\simeq$ 17 fold increase in maximum antimicrobial activity. Compared to the fed batch, the maximum activity was reached at 36 h instead of 33 h, with similar activity at 33 h. This indicates that substrate limitation contributes to the change in behavior from $r_P/q_P~>~r_{OX}$ to $r_P/q_P~<~r_{OX}$ and should therefore be avoided. Reducing substrate limitation resulted in a further $\simeq$ 1.2 fold increase in maximum antimicrobial activity over the previous best.

## Conclusion

**Fig. 6:**
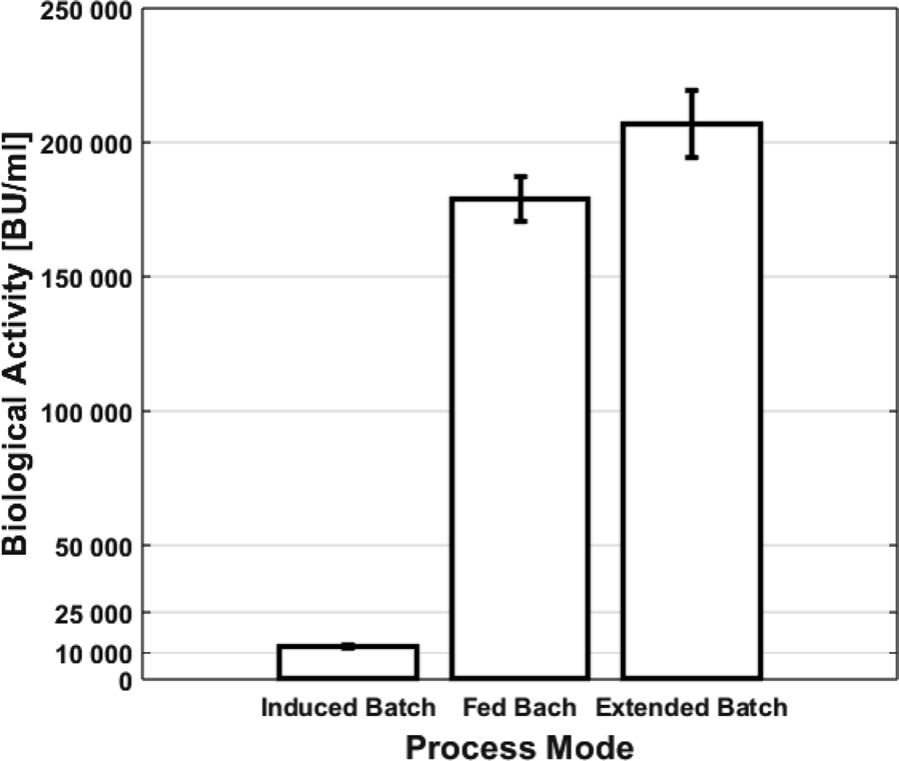
Maximum antimicrobial activity against *Listeria spp.* in biological units per ml cultivation supernatant for different process modes

Various process modes for the production of the antimicrobial peptide pediocin PA-1 with *C. glutamicum* on lignocellulosic minimal medium were compared (see Fig. 6). Induced batch processes with a single coupled growth and production phase resulted in up to $\simeq$ 6 mg/mL active pediocin PA-1. Fed batch processes up to $\simeq$ 90 mg/mL active pediocin PA-1, demonstrating that independently optimized conditions for growth and production result in significantly increased production. Extended batch processes produced up to $\simeq$ 104 mg/mL active pediocin PA-1, indicating that an unlimited substrate uptake regime during production results in an increase in production while reducing process complexity. In summary, the combination of a substrate uptake unlimited production regime and independently optimized growth and production phases was utilized to boost pediocin PA-1 production on cheap renewable carbon sources, exceeding even the highest previously reported production on yeast extract-based media by $\simeq$ 1.5 fold.

## Material and methods

### Strain and raw material

For this study, all experiments were performed using the strain *Corynebacterium glutamicum* CR099::U pXMJ19 - $\hbox {pedACD}^{Cg}$. This strain had genes encoding for improved mannose and xylose uptake integrated into the genome [29], as well as the genes required for production and secretion of pediocin PA-1 on a plasmid [15]. Biological activity of the pediocin PA-1 was tested against the *Listeria innocua* pNZ44 strain [15]. Low molecular weight permeate of ultra-filtered spent sulfite liquor (UF-SSL) obtained from Norway spruce (*Picea abies*) pulping (Borregaard AS, Sarpsborg, Norway) was used for all experiments. The UF-SSL was stored at 4 $^\circ$C. Within the UF-SSL, 4 metabolizable carbon sources were available at concentrations of 6.4 g/L acetate, 42 g/L glucose, 135 g/L mannose and 57 g/L xylose.

### Experimental setup

#### Preculture

Cells were stored at −80 $^\circ$C and streaked out onto 2TY-agar plates [16 g/L tryptone, 10 g/L yeast extract, 5 g/L NaCl, 10 g/L agar, heat sterilized, 12 mg/L chloramphenicol] and incubated [30 $^\circ$C, 48 h]. Afterwards, single colonies were used to inoculate 12.5 ml 2TY liquid medium (same composition as before but without agar) in 50-mL reaction tubes and incubated [30 $^\circ$C, 230 rpm, 24 h] as Seed 1. Subsequently, Seed 1 was transferred into 1-L shake flasks containing 225 mL 2TY medium and 25 ml SSL-MOPS [100 v/v% UF-SSL, 100 g/L 3-(N-morpholino)propanesulfonic acid (MOPS), pH 7.0, sterile filtered] and incubated [30 $^\circ$C, 230 rpm, 18 h] as Seed 2. For inoculation of the bioreactor, Seed 2 was harvested by centrifugation in 50-mL reaction tubes [3420 RCF, 4 $^\circ$C, 5 min] and resuspended in saline solution [0.9 g/L NaCl, heat sterilized] and 1 g/L cells were transferred to the bioreactor as inoculum using syringes (75 ml inoculum).

#### Bioreactor setup

*Lab-scale* bioreactors (Labfors 5, Infors, Germany) with 3-L working volume glass vessels equipped with optical $dO_2$ probes (Visiferm DO, Hamilton, Switzerland), potentiometric pH probes (Easyferm PHI, Hamilton, Switzerland) and off-gas analyzers (BlueInOne Ferm, BlueSense, Germany) were used. pH was controlled via the addition of 2.5 M KOH and 2.5 M $\hbox {H}_{2}\hbox {SO}_4$. $dO_2$ was controlled by increasing the agitator speed (400–1200 rpm) at a constant air flow of 0.3 vvm. Temperature was maintained at 30 $^\circ$C throughout the cultivation. Data were collected using a process information management system (PIMS) (Lucullus, SecureCell, Switzerland) and process control was performed using MATLAB (MATLAB2021b, Mathworks, USA) via the Rest-API interface of the PIMS. Sampling was performed every 3 h using an automated sampling device (custom built by the research group) and stored at 4 $^\circ$C until further analysis.

#### Cultivation conditions

A UF-SSL-based minimal medium for *C. glutamicum* including optimized batch growth conditions [17] were applied for this study with expansion to the production phase. As minimal medium [25 % UF-SSL, sterile filtered, 0.2 mg/L biotin, 12 mg/L chloramphenicol, 5 mL/L polyproylenglycol 2000] was used for cell growth and was further supplemented to a final concentration of 200 mM isopropyl-$\beta$-d-thiogalactopyranoside (IPTG) when recombinant production was desired. In the case of induced batch experiments, IPTG was present in the media from the start. IPTG was added via a syringe for fed batch and the feed was spiked with the final IPTG concentration. For extended batch cultivation’s, the feed was spiked with double the desired IPTG concentration and the syringe addition omitted. All experiments used a constant 200 mM IPTG as inducer throughout the production phase. Due to precipitation occurring upon mixing of UF-SSL with phosphate, a separate nitrogen and phosphorus feed (NP) was used [17]. For the batch, an NP feed with a carbon-to-nitrogen ratio (C/N) of 1:10, as reported to be optimal for growth of *C. glutamicum* by [30], and a carbon-to-phosphorous ratio (C/P) of 1:30, in order to ensure a constant bioavailable phosphorus supply, was used.

All bioreactor runs had a starting volume of 1 L minimal media, with 100 mL NP feed [66.4 g $\hbox {L}^{-1}$ urea and 71.8 g $\hbox {L}^{-1}$ $\hbox {KH}_{2}\hbox {PO}_{4}$, sterile filtered, $\dot{V}$ = 4.5 mL/h] added during the batch phase, resulting in a C/N ratio of 1:10 and a C/P ratio of 1:30. During both induced and uninduced batch phases a constant pH of 7.00 ± 0.02 and a $dO_2$ above 30 % was maintained. For fed batch and extended batch, cultivations were started after a fixed duration (24 h or 21 h, respectively) as indicated. 1 L minimal media feed was added for both fed batch and extended batch cultivations. Cultivation conditions were changed to pH between 5.5 and 7.0 and $dO_2$ of 15 % within ± 5 min from induction as indicated. A face-centered full-factorial design of experiment (DoE) was chosen with the C/P ratio (1:45/1:60/1:75) and the pH (6.0/6.5/7.0) as factors for the start after 24 h of batch phase (11 bioreactor experiments total). For fed batches started after 21 h of batch phase, 2 different design spaces (DS) were tested in a full-factorial designs with C/P ratio (1:30/1:45/1:60 & 1:45/1:60/1:75) and pH (5.5/6.0/6.5 & 6.0/6.5/7.0) as factors. Both feeds were controlled using the same exponential function [$\mu$ = 0.03 $\hbox {h}^{-1}$ $\simeq$ 25 % $\mu _{max}$] starting at the same time point [21/24 h as indicated] for the same duration [24 h] but with differing initial feed rates and feed volumes [C: $\hbox {V}_0$ = 1000 mL, $\dot{V_0}$ = 30 mL/h; NP: $\hbox {V}_0$ = 200 mL, $\dot{V_0}$ = 6 mL/h]. Thus, 1 P-mole was added for every *x* C-mole substrate (glucose, mannose, xylose, acetate) by adjusting the $\hbox {KH}_{2}\hbox {PO}_{4}$ concentration in the NP feed with a constant urea concentration. Extended batch cultivation’s were extended after 21 h of batch phase with a C/P of 1:45 and a pH of 6.0 during recombinant production (Table 1).

**Table 1:** Fed batch DoE DS overview

DoE	Factor 1 (C/P)	Factor 2 (pH)
21 h	1:45/1:60/1:75	6.0/6.5/7.0
24 h same DS	1:45/1:60/1:75	6.0/6.5/7.0
24 h adjusted DS	1:30/1:45/1:60	5.5/6.0/6.5

### Sample and data analysis

For *Off-line* analysis, samples were first centrifuged (3420 RCF, 4 $^\circ$C, 5 min) and the supernatant and pellet were separated. All data were evaluated using MATLAB (MATLAB 2021b, Mathworks, USA) and statistical analysis was performed using MODDE (MODDE 12.1, Sartorius AG, Germany).

#### Biomass

For the measurement of biomass in high water-insoluble solids (WIS) concentrations due to the phosphate precipitation, a cellular tryptophan-based fluorescence measurement was performed as described in [17]. Briefly, the pellet was resuspended and washed twice with saline solution before serial dilution on 96-well plates (PP black, Microplate, 96-well, F-Bottom; Greiner BIO-ONE) with saline solution. Subsequently, fluorescence (EX: 280/15 nm; EM: 340/20 nm) was measured using a plate reader (Spark, Tecan, Switzerland).

#### Pediocin PA-1

Biological activity of the antimicrobial activity (A) of the product (P) was measured by growth restriction of an indicator strain as described in [18]. The indicator strain was stored at −80 $^\circ$C and incubated (37 $^\circ$C, overnight) in BHI medium [37.5 g/L BHI (Oxoid Brain-Heart-Infusion, Thermo Fisher Scientific, USA), heat sterilized, 12 mg/L chloramphenicol]. A serial dilution of the supernatant in fresh BHI medium on a 96-well plate (CELLSTAR, sterile Microplate, 96-well, F-Bottom; Grainer BIO-ONE) was performed, BHI medium with grown indicator strain added 1:1 and subsequently incubated (37 $^\circ$C, 5–6 h). Thereafter, optical density as 600 nm ($OD_{600}$) was measured using a plate reader (Spark, Tecan, Switzerland). In order to generate more precise biological activity data using the 1:2 serial dilution of the original assay, a dosage–response curve was used as described in [21] with a non-linear iterative least square algorithm. The adapted dosage–response curve (Eq. 1), calculates the optical density $OD_{600}$ at 50 % growth inhibition from the lower bound $LB_{OD_{600}}$, taken from the dilution with the lowest $OD_{600}$, the upper bound $UB_{OD_{600}}$, taken from the blank, the inflection point $ID_{50}$, the critical dilution *D* and the curvature *k*, with an illustration provided in Fig. 1. The biological activity measured in biological units (BU$\cdot$m$\hbox {L}^{-1}$) was defined as the pediocin PA-1 concentration at which the growth of the indicator strain was reduced to 50 % of the uninhibited growth and calculated from the infection points ($\hbox {ID}_{50}$) of the fitted curve:

1 $$\begin{aligned} OD_{600} = LB_{OD_{600}} \; + \; \frac{UB_{OD_{600}} \; - \; LB_{OD_{600}}}{1 \; + \; (ID_{50}/D)^k}. \end{aligned}$$

## Supplementary Information

Supplementary Material 1

## Data Availability

The datasets generated and/or analyzed during the current study are available in the Mendeley Data repository, DOI: 10.17632/8d6skttnbf.1.

## Abbreviations

ABE: Acetone, butanol, ethanol fermentation
AMP: Antimicrobial peptides
CER: Carbon dioxide evolution rate
C/N: Carbon-to-nitrogen ratio
C/P: Carbon-to-phosphate ratio
DoE: Design of experiment
DS: Design space
EFSA: European Food Safety Administration
EU: European Union
GMO: Genetically modified organism
HPLC: High-pressure liquid chromatography
IPTG: Isopropyl-$\beta$-d-thiogalactopyranoside
LBM: Lignocellulosic biomass
MLR: Multiple linear regression
MOPS: 3-(N-morpholino)propanesulfonic acid
NP: Nitrogen and phosphorus
OUR: Oxygen uptake rate
PIMS: Process information management system
SSL: Spent sulfite liquor
SCP: Single cell protein
UF-SSL: Ultra-filtered spent sulfite liquor
WIS: Water-insoluble solids

## References

[1] Usmani Z, Sharma M, Awasthi AK, Lukk T, Tuohy MG, Gong L, et al. Lignocellulosic biorefineries: the current state of challenges and strategies for efficient commercialization. Renew Sustain Energy Rev. 2021;148: 111258.

[2] Hassan SS, Williams GA, Jaiswal AK. Moving towards the second generation of lignocellulosic biorefineries in the EU. Renew Sust Energ Rev. 2019;101:590–9.

[3] Garcia-Ochoa F, Vergara P, Wojtusik M, Gutiérrez S, Santos VE, Ladero M, et al. Multi-feedstock lignocellulosic biorefineries based on biological processes: an overview. Ind Crops Prod. 2021;172: 114062.

[4] Laluce C, Schenberg ACG, Gallardo J, Coradello L, Pombeiro-Sponchiado S. Advances and developments in strategies to improve strains of *Saccharomyces cerevisiae* and processes to obtain the lignocellulosic ethanol- A review. Appl Biochem Biotechnol. 2012;166:1908–26. 22391693 10.1007/s12010-012-9619-6

[5] Brethauer S, Wyman CE. Continuous hydrolysis and fermentation for cellulosic ethanol production. Biores Technol. 2010;101(13):4862–74. 10.1016/j.biortech.2009.11.009 20006926

[6] Kosamia NM, Samavi M, Piok K, Rakshit SK. Perspectives for scale up of biorefineries using biochemical conversion pathways: technology status, techno-economic, and sustainable approaches. Fuel. 2022;324: 124532.

[7] Halme A, Holmberg A, Tiussa E. Modelling and control of a protein fermentation process utilizing the spent sulphite liquor. IFAC Proc Vol. 1977;10(7):817–25.

[8] Gold D, Mohagheghi A, Cooney CL, Wang DI. Single-cell protein production from spent sulfite liquor utilizing cell-recycle and computer monitoring. Biotechnol Bioeng. 1981;23(9):2105–16.

[9] Lo S, Moreau J. Mixed-culture microbial protein from waste sulfite pulping liquor II: its production on pilot-plant scale and use in animal feed. Can J Chem Eng. 1986;64(4):639–46.

[10] Fischer S, Titgemeyer F. Protective cultures in food products: from science to market. Foods. 2023;12:1541. 37048362 10.3390/foods12071541 PMC10094266

[11] on Additives EP, or Substances used in Animal Feed (FEEDAP) P. Scientific Opinion on the safety and efficacy of lignosulphonate as a feed additive for all animal species. EFSA J. 2015;13(7):4160. 10.2903/j.efsa.2015.4160 PMC13151553 42109796

[12] Cankar K, Henke NA, Wendisch VF. Functional food additives/ingredients production by engineered *Corynebacterium glutamicum*. Syst Microbiol Biomanuf. 2023;3(1):110–21.

[13] Weixler D, Berghoff M, Ovchinnikov KV, Reich S, Goldbeck O, Seibold GM, et al. Recombinant production of the lantibiotic nisin using *Corynebacterium glutamicum* in a two-step process. Microb Cell Fact. 2022;21(1):11. 35033086 10.1186/s12934-022-01739-y PMC8760817

[14] Desiderato CK, Hasenauer KM, Reich SJ, Goldbeck O, Holivololona L, Ovchinnikov KV, et al. Garvicin Q: characterization of biosynthesis and mode of action. Microb Cell Fact. 2022;21(1):236. 36368990 10.1186/s12934-022-01952-9 PMC9652874

[15] Goldbeck O, Desef DN, Ovchinnikov KV, Perez-Garcia F, Christmann J, Sinner P, et al. Establishing recombinant production of pediocin PA-1 in *Corynebacterium glutamicum*. Metab Eng. 2021;68:34–45. 34492380 10.1016/j.ymben.2021.09.002 PMC8593837

[16] Becker J, Rohles CM, Wittmann C. Metabolically engineered *Corynebacterium glutamicum* for bio-based production of chemicals, fuels, materials, and healthcare products. Metab Eng. 2018;50:122–41. 30031852 10.1016/j.ymben.2018.07.008

[17] Waldschitz D, Neudert MR, Kitzmueller J, Lachmann J, Fonteyne A, Maes K, et al. Robust, fully quantifiable and scalable bioprocess utilizing spent sulfite liquor with *Corynebacterium glutamicum*. Bioresour Technol. 2024;130967. 10.1016/j.biortech.2024.130967 38880268

[18] Ovchinnikov KV, Oftedal TF, Reich SJ, Bar NS, Holo H, Skaugen M, et al. Genome-assisted identification, purification, and characterization of bacteriocins. Bio-Protoc. 2022;12(14):e4477–e4477. 35978579 10.21769/BioProtoc.4477 PMC9350922

[19] Christmann J, Cao P, Becker J, Desiderato CK, Goldbeck O, Riedel CU, et al. High-efficiency production of the antimicrobial peptide pediocin PA-1 in metabolically engineered *Corynebacterium glutamicum* using a microaerobic process at acidic pH and elevated levels of bivalent calcium ions. Microb Cell Fact. 2023;22(1):1–18. 36849884 10.1186/s12934-023-02044-y PMC9969654

[20] Mandenius CF, Brundin A. Bioprocess optimization using design-of-experiments methodology. Biotechnol Prog. 2008;24(6):1191–203. 19194932 10.1002/btpr.67

[21] Di Veroli GY, Fornari C, Goldlust I, Mills G, Koh SB, Bramhall JL, et al. An automated fitting procedure and software for dose-response curves with multiphasic features. Sci Rep. 2015;5(1):14701. 26424192 10.1038/srep14701 PMC4589737

[22] Frost J. Regression analysis: an intuitive guide for using and interpreting linear models. Statisics By Jim Publishing; 2019.

[23] Wilcox RR. Estimation in the simple linear regression model when there is heteroscedasticity of unknown form. Commun Stat Theory Methods. 1996;25(6):1305–24.

[24] Leslie DS, Kohn R, Nott DJ. A general approach to heteroscedastic linear regression. Stat Comput. 2007;17:131–46.

[25] Fatehi P, Ni Y. Integrated forest biorefinery- sulfite process. In: Sustainable production of fuels, chemicals, and fibers from forest biomass. ACS Publications; 2011. p. 409–441.

[26] Becker N, Lebo S. Recovery of proteins by preciptation using lignosulfonates. US Patent App. 09/918771.

[27] Jack Z, Cairney TJ. Lignin-bacitracin complex as growth stimulant and bacitracin purifier. US Patent 3035919.

[28] Jakob K, Satorhelyi P, Lange C, Wendisch VF, Silakowski B, Scherer S, et al. Gene expression analysis of *Corynebacterium glutamicum* subjected to long-term lactic acid adaptation. J Bacteriol. 2007;189(15):5582–90. 17526706 10.1128/JB.00082-07 PMC1951826

[29] Cao P, Christmann J, Schwechheimer S, Becker J, Wittmann C. Utilization of spent sulphite liquor by *Corynebacterium glutamicum*. (Manuscript in preparation). 2024;.

[30] Kiefer D, Merkel M, Lilge L, Hausmann R, Henkel M. High cell density cultivation of *Corynebacterium glutamicum* on bio-based lignocellulosic acetate using pH-coupled online feeding control. Bioresour Technol. 2021;340: 125666. 34352645 10.1016/j.biortech.2021.125666

