# Supplementary material for: Antimicrobial peptide production with Corynebacterium glutamicum on lignocellulosic side streams
Source: Biotechnol Biofuels Bioprod. 2024 Dec 18;17:147. doi: 10.1186/s13068-024-02587-1 (PMC11657226; doi:10.1186/s13068-024-02587-1)
Supplement: Supplementary file 1 — Supplementary Material 1 [file 13068_2024_2587_MOESM1_ESM.docx]

**Antimicrobial peptide production with *Corynebacterium glutamicum* on lignocellulosic side streams**

Daniel Waldschitz, Mark-Richard Neudert, Jakob Kitzmüller, Yannick Bus, Eva Maria Karner, Jae Hwi Bong, Peter Sinner, Oliver Spadiut

**MLR model statistics**

Table 1: Statistical data fort he MLR model of the 24h DoE as provided by the used Modde 13.0 Pro Software package.

| Amax | DF | SS | MS (variance) | F | p | SD |
| --- | --- | --- | --- | --- | --- | --- |
| Total | 12 | 8.80643e+09 | 7.33869e+08 |  |  |  |
| Constant | 1 | 5.60512e+09 | 5.60512e+09 |  |  |  |
|  |  |  |  |  |  |  |
| Total corrected | 11 | 3.20131e+09 | 2.91028e+08 |  |  | 17059.6 |
| Regression | 5 | 3.01027e+09 | 6.02054e+08 | 18.9085 | **0.001** | 24536.8 |
| Residual | 6 | 1.91042e+08 | 3.18404e+07 |  |  | 5642.73 |
|  |  |  |  |  |  |  |
| Lack of Fit | 3 | 1.03712e+08 | 3.45707e+07 | 1.18759 | **0.445** | 5879.68 |
| (Model error) |  |  |  |  |  |  |
| Pure error | 3 | 8.73301e+07 | 2.911e+07 |  |  | 5395.37 |
| (Replicate error) |  |  |  |  |  |  |
|  |  |  |  |  |  |  |
|  | N = 12 | Q2 = | 0.778 | Cond. no. = | 3.355 |  |
|  | DF = 6 | R2 = | 0.940 | RSD = | 5643 |  |
|  |  | R2 adj. = | 0.891 |  |  |  |

**Lignosulfonate-Peptide complex precipitation**


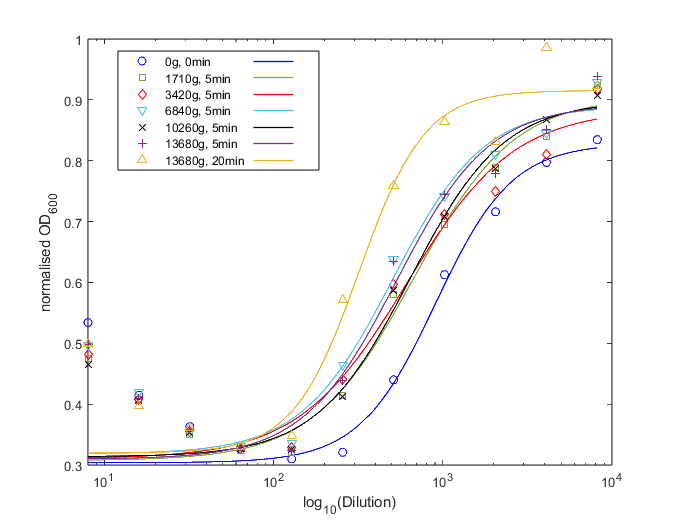


a)


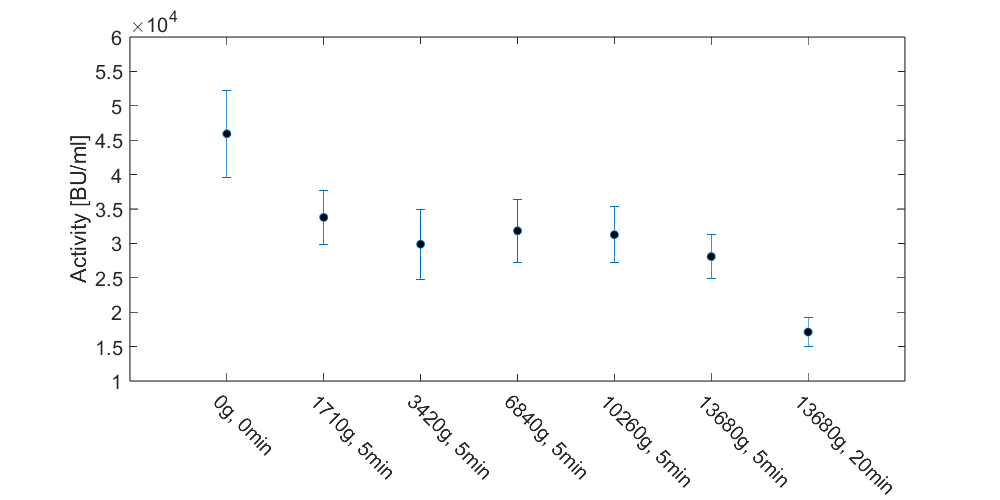


b)

Figure 1: Pediocin PA-1 standard mixed with 25% UF-SSL and spun down at different speeds and durations at pH 4.3-4.5 (native pH of the UF-SSL used), a) Differences in the curve fit (shown for one replicate) are observable for no centrifugation as well as centrifugation at max stings for longer periods. Higher normalized OD values at lower Dilution are a result of matrix components such as sugars which can increase growth of the indicator organism, as well as the peptide-lignosulfonate complex formation. The effect diminishes at higher Dilution, reducing the influence of matric components at higher pediocin PA-1 concentrations. b) The calculated activity (in biological triplicates) follows the same trend as the curve fit, showing initial peptide precipitation at all settings while for more quantitative precipitation longer centrifugation durations are necessary. 3420g, 5min were used for all experiments as cell separation had to be performed and loss free operation conditions were not feasible. Since the peptide-lignosulfonate complex formation is reported to be broken at pH over the protein pI (8.8 for pediocin PA-1) the native pH of UF-SSL represents the worst case with the strongest complex binding (all experiments at higher pH), which complex bonding strength being indistinguishable from other pH-based effects on production.

**Influence of Media components on antimicrobial assay**


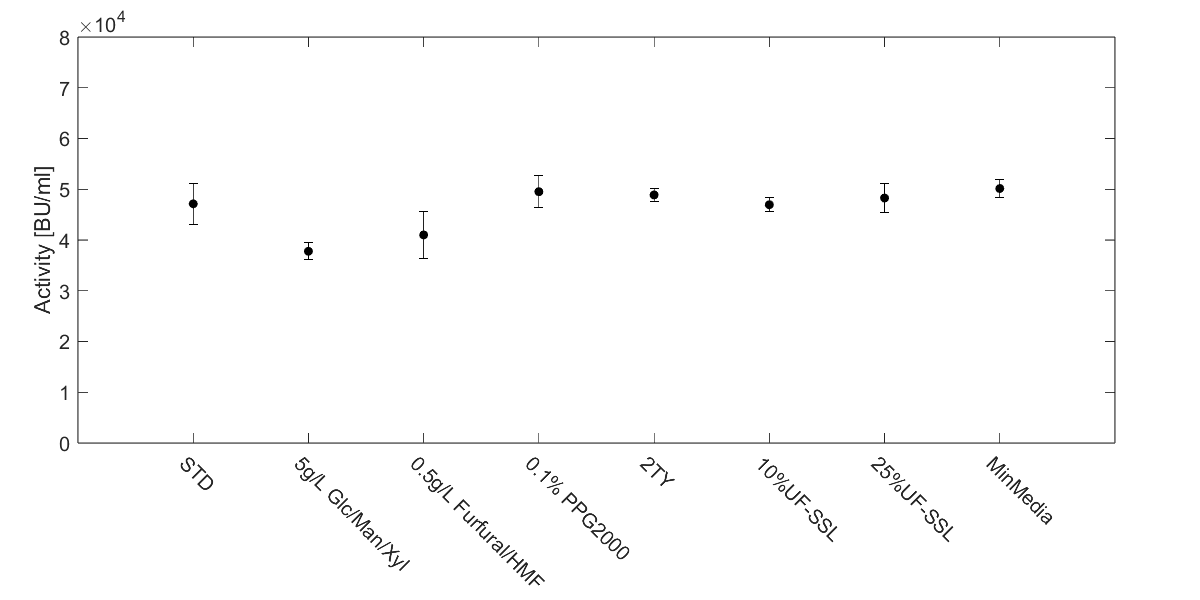
 Figure 2: Pediocin PA-1 standard mixed with (to an end concentration of) dH_2_O; 5g/L glucose, mannose and xylose; 0.5 g/L furfural and 5-Hydroxymethlyfurfural; 0.1% Polypropylene glycol 2000; 2TY yeast extract medium as used for reference studies; 10% UF-SSL; 25%% UF-SSL; UF-SSL based minimal medium after cultivation without induction. Error bars indicate the spread of biological triplicates in the antimicrobial assay calculated from the results after the curve fit.

**Oxidation of Pediocin PA-1 in different media**

1. Oxidation of Pediocin PA-1 standard in 2TY medium with H_2_O_2_


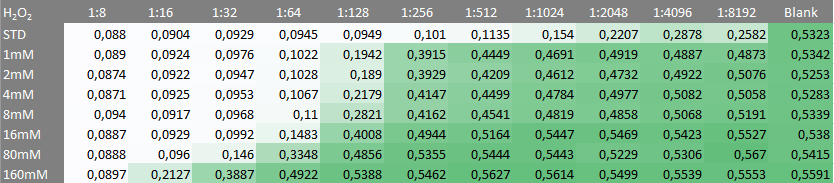


1. Oxidation of Pediocin PS-1 standard in 25%UF-SSL medium with H_2_O_2_


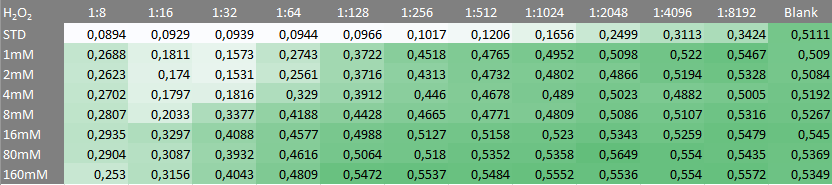


Figure 3: Pediocin PA-1 standard in 2TY yeast extract based medium used for reference studies (top) and 25% UF-SSL (bottom) oxidized using 1-160mM H_2_O_2_ at RT for 1h before measurement of the antimicrobial activity assay. Data values shown represent the OD600 of the grown indicator organism after 6h of incubation at 37°C. The loss of activity due to oxidizing agents was found to be higher in UF-SSL based medium then yeast extract based medium.
